# Association Between Temporal Muscle Thickness and Skeletal Muscle Mass, Nutritional Status, and Physical Function in Patients With Post-stroke Hemiparesis: A Cross-Sectional Study

**DOI:** 10.7759/cureus.86253

**Published:** 2025-06-17

**Authors:** Shunmei Terui, Motoki Maruyama, Manabu Horikawa, Emi Oku, Yusei Kiyota, Masahiro Sasaki, Hiroaki Shimizu

**Affiliations:** 1 Rehabilitation, Akita Cerebrospinal and Cardiovascular Center, Akita, JPN; 2 Radiology, Akita Cerebrospinal and Cardiovascular Center, Akita, JPN; 3 Rehabilitation Medicine, Akita Cerebrospinal and Cardiovascular Center, Akita, JPN

**Keywords:** nutritional status, sarcopenia, skeletal muscle index, stroke rehabilitation, temporal muscle thickness

## Abstract

Introduction

Temporal muscle thickness (TMT), observed on brain magnetic resonance imaging (MRI), has emerged as a potential surrogate marker for sarcopenia. This study aimed to investigate the relationships between TMT, muscle mass, nutritional status, and lower limb motor function in individuals with post-stroke hemiparesis.

Methods

This cross-sectional study enrolled 45 patients experiencing their first-ever stroke with hemiparesis, all of whom were discharged from a rehabilitation ward. TMT was assessed using T2-weighted MRI images. Muscle mass and quality were evaluated using the skeletal muscle index (SMI), extracellular water-to-total body water ratio (ECW/TBW), and phase angle (PhA). Nutritional status was determined by serum albumin levels, the Geriatric Nutritional Risk Index (GNRI), and Body Mass Index (BMI). Lower limb function was assessed using the Stroke Impairment Assessment Set Motor Function of the Lower Extremities (SIAS-MLE). Analyses were conducted using Spearman’s correlation and intraclass correlation coefficients (ICCs).

Results

TMT demonstrated high inter-rater reliability (ICC: 0.812 for the left side; 0.796 for the right). Positive correlations were observed between TMT and SMI, PhA, GNRI, and SIAS-MLE. A negative correlation was found with ECW/TBW. No significant correlations were identified with age, BMI or handgrip strength.

Conclusion

TMT is a reliable and practical marker that correlates with muscle mass, muscle quality, nutritional status, and motor function in patients post-stroke. These findings underscore its potential role in sarcopenia screening.

## Introduction

Sarcopenia is characterized by a decline in skeletal muscle mass and strength associated with aging, which results in decreased physical function [1]. It has also been shown to negatively affect recovery and prognosis in patients with post-stroke hemiparesis [2]. The standard diagnostic criteria for sarcopenia include the assessment of muscle mass, handgrip strength, and gait speed [3,4]. However, in patients with stroke, functional assessments such as handgrip strength and gait speed may not be feasible due to impaired consciousness, prolonged bed rest, or motor paralysis [5].

Common methods for measuring skeletal muscle mass include dual-energy X-ray absorptiometry (DXA) and bioelectrical impedance analysis (BIA) [6,7]. Although DXA provides high accuracy, it requires specialized and costly equipment. BIA is more widely available and easier to use, but is affected by the patient’s hydration status, which reduces reliability in the presence of edema or dehydration. Therefore, the assessment of sarcopenia in patients with stroke is constrained by patient-related limitations and limited equipment availability.

Recently, temporal muscle thickness (TMT) has emerged as a potential alternative marker for skeletal muscle mass and a predictor of functional outcomes in patients with stroke [8-10]. The temporalis muscle, a masticatory muscle, is one of the few skeletal muscles consistently visible on routine neuroimaging, such as head computed tomography (CT) and magnetic resonance imaging (MRI). If TMT reliably reflects skeletal muscle mass, it could make use of existing neuroimaging data without the need for additional scans or specialized equipment.

However, only a limited number of studies have examined the relationship between TMT and nutritional status, in addition to muscle mass. Patients with stroke are at risk of malnutrition due to factors such as dysphagia and decreased physical activity [11], which may contribute to the progression of sarcopenia and worsen functional outcomes [12]. Therefore, evaluating whether TMT can serve as an indicator of nutritional status, as well as muscle mass, is clinically important.

This study aimed to investigate the relationship between TMT, skeletal muscle mass, and nutritional markers in patients with post-stroke hemiparesis, and to evaluate the potential role of TMT as a tool for assessing sarcopenia and nutritional status.

## Materials and methods

Study design and participants

This study employed a cross-sectional design. Consecutive patients with first-ever stroke and hemiparesis, admitted to the convalescent rehabilitation ward at our center between October 2021 and October 2022, were screened for inclusion.

Patients were excluded if they (1) had undergone craniotomy, (2) had pacemakers or other metallic implants that contraindicated MRI, (3) had MRI scans with motion artifacts that compromised reliable TMT measurement, or (4) were unable to complete the assessments prior to discharge.

Data collection

Basic demographic and clinical information, including age, sex, Body Mass Index (BMI), length of stay, and lower limb motor function measured by the Stroke Impairment Assessment Set Motor Function of the Lower Extremities (SIAS-MLE), was collected from electronic medical records. Handgrip strength was assessed using a Smedley-type dynamometer, with two measurements taken on each hand. The highest value was used for analysis.

TMT measurement

TMT was measured using T2-weighted images acquired at admission and discharge with a 3-Tesla MRI scanner (Magnetom Verio; Siemens Healthineers, Erlangen, Germany). Two evaluators conducted the measurements: one physical therapist with 10 years of clinical experience, and one radiologic technologist with 12 years of experience. A picture archiving and communication system (Synapse PACS; Fujifilm Medical, Tokyo, Japan) was used for analysis.

**Figure 1:**
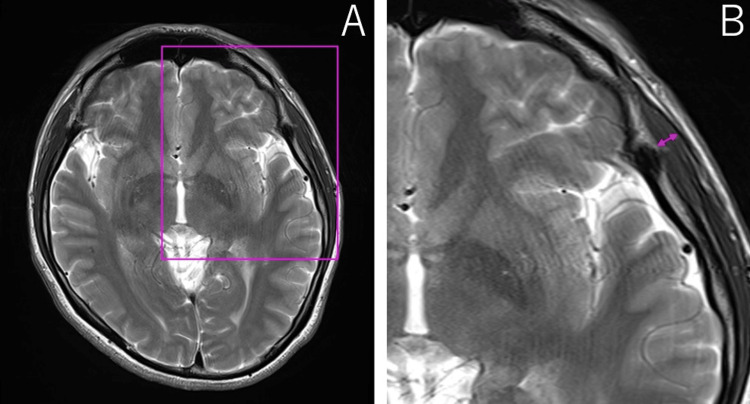
TMT assessment represented on cranial T2-weighted contrast-enhanced MRIs (A) Axial T2-weighted image obtained at the level of the orbital apex. (B) The supraorbital fissure and orbital apex were used as anatomical landmarks. The Sylvian fissure was identified as a reference point for the anterior-posterior orientation. TMT was measured bilaterally, perpendicular to the long axis of the muscle. TMT, temporal muscle thickness; MRI, magnetic resonance imaging

TMT was measured according to a previously reported method [13,14], using the supraorbital fissure and orbital apex as anatomical landmarks. At the level of the orbital apex, the thickness of the temporalis muscles on the right and left sides was measured perpendicular to their long axis (Figure 1). The average of the right and left TMT values was calculated for each patient. All measurements were performed by a board-certified radiologist who was blinded to the patients’ clinical information.

Assessment of muscle mass and quality

Skeletal muscle mass was evaluated using a body composition analyzer, InBody S10 (InBody Japan, Inc., Tokyo, Japan), with measurements taken in the supine position after ensuring adequate rest. The skeletal muscle index (SMI) served as an indicator of skeletal muscle mass. Recently, diagnostic criteria for sarcopenia have emphasized the importance of assessing not only muscle mass but also muscle quality [15,16]. Therefore, in addition to SMI, the extracellular water-to-total body water ratio (ECW/TBW) and phase angle (PhA) were measured as markers of muscle quality.

Assessment of nutritional status

Nutritional status was assessed using serum albumin and the Geriatric Nutritional Risk Index (GNRI) [17], based on pre-discharge blood test results. GNRI was calculated using serum albumin, actual body weight, and ideal body weight according to the following formula: $14.89 \times \text{serum albumin (g/dL)} + 41.7 \times \frac{\text{actual body weight (kg)}}{\text{ideal body weight (kg)}}$.

Ideal body weight was calculated as height (m)² × 22, corresponding to a BMI of 22. If the actual body weight exceeded the ideal body weight, the weight ratio was set to 1.

Statistical analysis

Inter-rater reliability of TMT was assessed using the intraclass correlation coefficient (ICC) (2,1), with a 95% confidence interval (CI).

The relationships between TMT and eight variables (age, sex, BMI, SMI, ECW/TBW, GNRI, SIAS-MLE, and handgrip strength) were examined using Spearman’s rank correlation coefficient (two-tailed). All analyses were performed using R version 4.3.1 (R Foundation for Statistical Computing, Vienna, Austria). A p-value of <0.05 was considered statistically significant.

Ethics

This study received approval from the Ethics Committee of the Akita Cerebrospinal and Cardiovascular Center (approval date: July 27, 2021; approval number: 21-16). Participants were provided written information explaining their right to withdraw from the study at any time (opt-out) without any disadvantages. Personal information was managed with strict confidentiality.

## Results

**Figure 2:**
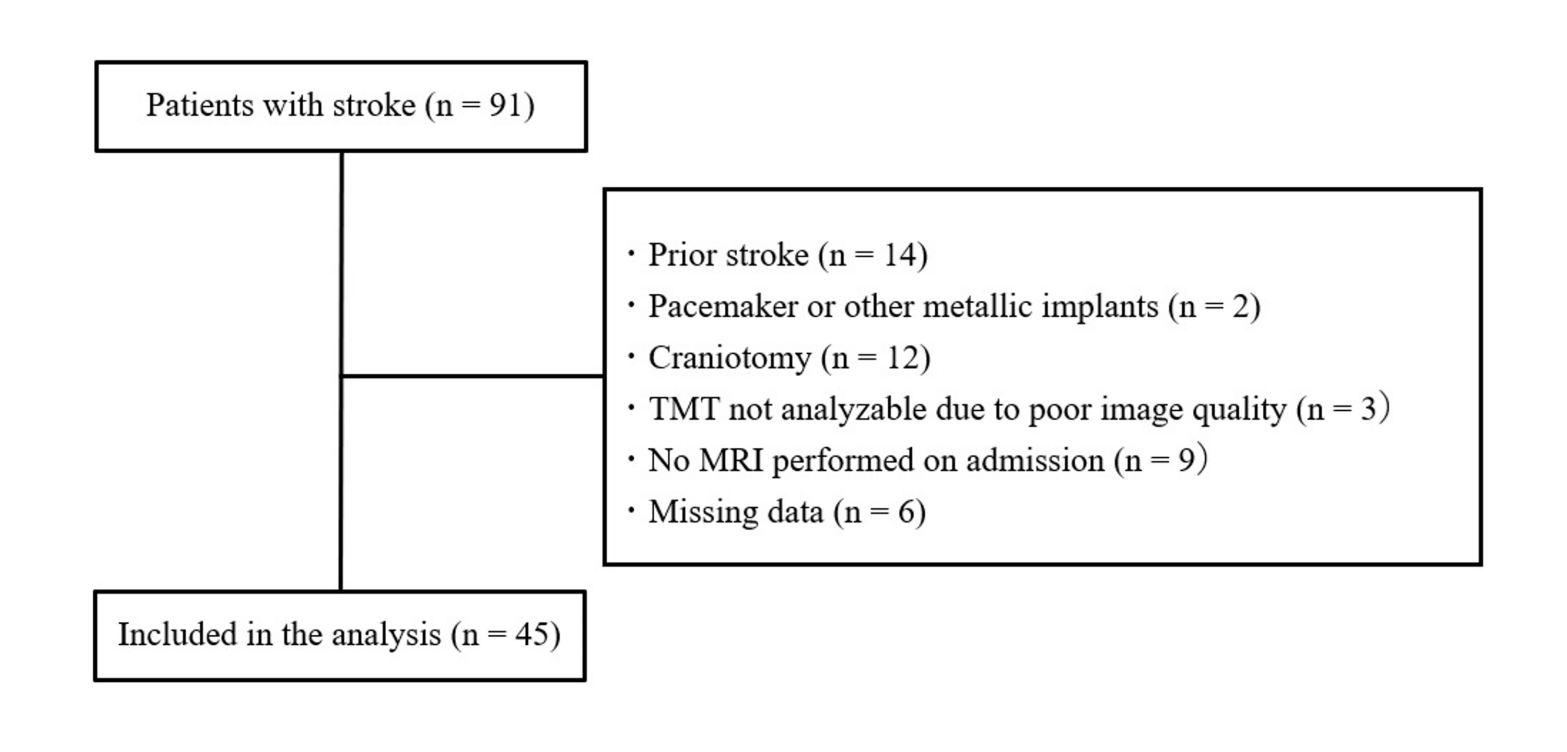
Flowchart of participant exclusion

Out of 91 stroke patients screened, 46 were excluded for the following reasons: prior stroke (n = 14), presence of a pacemaker or other metallic implants (n = 2), history of craniotomy (n = 12), poor-quality MRI images unsuitable for TMT measurement (n = 3), no MRI performed at admission (n = 9), or missing data (n = 6). Therefore, 45 patients were included in the final analysis (Figure 2).

Table 1 shows baseline characteristics by sex. The median age was 67.8 years (interquartile range (IQR): 59.5-75.6). Stroke subtypes included cerebral infarction in 21 patients (52.5%), cerebral hemorrhage in 18 patients (45.0%), and subarachnoid hemorrhage in one patient (2.5%). The mean GNRI score was 96.8 ± 7.6. Significant differences between males and females were found in handgrip strength (p < 0.001), TMT (p = 0.045), and SMI (p < 0.001). The mean handgrip strength was 31.6 ± 9.7 kg for males and 18.8 ± 7.8 kg for females. Mean TMT was 5.3 ± 1.5 mm in males and 4.9 ± 0.9 mm in females. Mean SMI was 6.8 ± 0.8 kg/m² in males and 5.7 ± 0.7 kg/m² in females. No significant differences were observed in age, BMI, SIAS-MLE, ECW/TBW, serum albumin, or GNRI.

**Table 1:** Characteristics of each patient by sex a, Mann-Whitney U test; b, t-test; c, Fisher’s exact test SD, standard deviation; IQR, interquartile range; SISAS-MLE, stroke impairment assessment set motor function of the lower extremities; TMT, temporal muscle thickness; SMI, skeletal muscle mass index; ECW/TBW, extracellular water-to-total body water ratio; GNRI, geriatric nutritional risk index

Variables	Total (N = 40)	Men (N = 26)	Women (N = 14)	p
Age, years (IQR)	67.8 (59.5-75.6)	67.9 (59.0-75.5)	67.7 (62.5-75.0)	0.943^a^
BMI, kg/m^2^ (SD)	22.5 (4.9)	22.1 (5.6)	22.8 (3.8)	0.552^b^
Stroke subtype, n (%)				0.374^c^
Cerebral infarction	21 (52.5)	13 (50.0)	8 (57.1)	
Cerebral hemorrhage	18 (45.0)	13 (50.0)	5 (35.7)	
Subarachnoid hemorrhage	1 (2.5)	0 (0.0)	1 (7.1)	
Length of stay (IQR)	106 (81-133)	107 (85-131)	103 (81-133)	0.854^a^
SIAS-MLE, score (IQR)	11 (9-15)	11 (10-15)	11 (9-15)	0.814^a^
Handgrip strength, kg (SD)	26.9 (10.1)	31.6 (9.7)	18.8 (7.8)	<0.001^b^
TMT, mm (SD)	5.0 (1.4)	5.3 (1.5)	4.9 (0.9)	0.045^b^
SMI, kg/m^2^ (SD)	6.4 (0.9)	6.8 (0.8)	5.7 (0.7)	<0.001^b^
ECW/TBW, % (SD)	0.396 (0.1)	0.4 (0.1)	0.4 (0.1)	0.071^b^
Serum albumin, g/dL (SD)	3.8 (0.4)	3.8 (0.4)	3.8 (0.4)	0.781^b^
GNRI, score (SD)	96.8 (7.6)	96.3 (8.2)	97.8 (6.6)	0.552^b^

The ICC (2,1) for mean TMT between raters was 0.812 (95% CI: 0.672-0.896) on the left side and 0.796 (95% CI: 0.706-0.919) on the right side (Table 2).

**Table 2:** Inter-rater reliability of TMT measurements TMT, temporal muscle thickness; ICC, intraclass correlation coefficient; CI, confidence interval

		ICC (2,1)		ICC (2,2)	
		Average	95% CI	Average	95% CI
TMT	Left	0.786	0.601-0.885	0.881	0.600-0.885
	Right	0.796	0.660-0.882	0.883	0.659-0.883

Spearman’s correlation analysis revealed that TMT was positively correlated with SIAS-MLE (ρ = 0.468, p = 0.0023), SMI (ρ = 0.396, p = 0.0114), PhA (ρ = 0.528, p < 0.001), and GNRI (ρ = 0.350, p = 0.0267). A negative correlation was found between TMT and ECW/TBW (ρ = -0.460, p = 0.0028). No significant correlations were detected between TMT and age, BMI, length of stay, or handgrip strength (Table 3).

**Table 3:** Correlation between TMT and clinical variables BMI, body mass index; SIAS-MLE, stroke impairment assessment set motor function of the lower extremities; SMI, skeletal muscle index; ECW/TBW, extracellular water-to-total body water ratio; PhA, phase angle; GNRI, geriatric nutritional risk index; ρ, Spearman’s correlation coefficient; CI, confidence interval; p, p-value

	ρ	95% CI	p
Age, years	-0.004	-0.315, 0.308	0.9816
BMI, kg/m^2^	0.293	-0.021, 0.554	0.06666
Length of stay, days	-0.223	-0.500, 0.095	0.1665
SIAS-MLE, score	0.468	0.183, 0.681	0.002306
SMI, kg/m^2^	0.396	0.097, 0.630	0.01135
Handgrip strength, kg	0.306	-0.006, 0.564	0.05442
ECW/TBW, %	-0.460	-0.675, -0.173	0.002848
PhA, °	0.528	0.259, 0.721	<0.001
GNRI, score	0.350	0.044, 0.579	0.02669

## Discussion

In this study, we showed that TMT measured by MRI in patients with post-stroke hemiparesis was significantly associated with skeletal muscle mass, muscle quality, nutritional status, and SIAS-MLE, and that it demonstrated high inter-rater reliability. These results suggest that TMT could be a valuable marker for evaluating sarcopenia and predicting functional outcomes in this patient group.

TMT measurements conducted by a physical therapist and a radiologic technologist achieved high inter-rater reliability. This suggests that even physical therapists without routine experience interpreting MRI scans can reliably measure TMT. Moreover, it indicates that TMT can be assessed with acceptable accuracy, without requiring specialized radiological expertise, supporting its potential use in rehabilitation settings. This may improve the practicality of sarcopenia assessment in clinical rehabilitation.

TMT showed positive correlations with SIAS-MLE, SMI, GNRI, and muscle quality indices (PhA and ECW/TBW). These results suggest that TMT reflects not only muscle mass but also muscle quality and nutritional status. Patients with post-stroke hemiparesis commonly experience reductions in muscle mass and quality due to paralysis and decreased activity [18]; therefore, TMT may serve as a noninvasive marker of these changes. However, because TMT measures the thickness of a localized muscle, further research is necessary to clarify its association with whole-body muscle quality, other muscle groups, and clinical outcomes.

The positive correlation between TMT and SIAS-MLE suggests that TMT may relate to lower limb function and mobility, extending beyond local muscle mass. In patients with post-stroke hemiparesis, lower limb muscle strength decreases as a result of paralysis and disuse [19], and TMT may reflect this decline. In addition, severe strokes are often associated with a higher prevalence of dysphagia [20,21], which leads to reduced masticatory function and subsequent atrophy of the temporalis muscle, contributing to lower TMT. Thus, TMT may represent masticatory dysfunction and nutritional decline, warranting further studies to investigate its connection to swallowing function.

The significant positive correlation between TMT and GNRI further supports the use of TMT as a marker of nutritional status. In contrast, previous studies have reported no such association [22]. This difference may be explained by the timing of measurements. Earlier studies assessed TMT at the time of stroke onset, whereas we measured TMT at discharge - a time when temporalis atrophy may have progressed due to impaired consciousness, paralysis, or dysphagia.

This study has several limitations. First, as a single-center study with a small sample size, the findings have limited generalizability and should be interpreted with caution. Second, the cross-sectional design precludes causal inference between TMT and the variables examined. Third, swallowing function was not evaluated in detail, which limits understanding of its relationship with TMT. Future research should include larger, multicenter, longitudinal, and multivariate analyses to better assess the clinical use of TMT.

## Conclusions

In conclusion, the high inter-rater reliability of TMT measurements between physical therapists and radiologic technologists suggests that TMT can be consistently assessed even by non-specialists, supporting its practical use in rehabilitation settings. Our findings further indicate that TMT may serve as a valuable indicator for comprehensively evaluating sarcopenia, nutritional status, and physical function in patients with post-stroke hemiparesis.
